# Perspective: Total, Added, or Free? What Kind of Sugars Should We Be Talking About?

**DOI:** 10.1093/advances/nmx020

**Published:** 2018-04-07

**Authors:** David J Mela, Elizabeth M Woolner

**Affiliations:** 1 Unilever R&D Vlaardingen, Vlaardingen, Netherlands; 2 Unilever R&D Colworth, Sharnbrook, United Kingdom

**Keywords:** sugar, health, recommendations, labeling, regulation

## Abstract

There is consistent public guidance to limit sugars intakes. However, WHO recommendations are for “free” sugars, whereas some other guidance documents and public discussion focus on “added” sugars, and globally most food labeling states “total” sugars. Total sugars comprise all mono- and disaccharides, regardless of source, whereas both added and free sugars exclude the sugars that naturally occur in dairy products and intact fruit and vegetables. Definitions of added and free sugars differ mainly in their respective exclusion or inclusion of sugars in juiced or pureed fruit and vegetables. To date, there has been little evidence-based analysis of the scientific basis for these different sugar classifications or implications of their adoption for consumer communication and nutrition labeling. Evidence of discriminating relations of total compared with added or free sugars with weight gain or energy intake, type 2 diabetes, and dental caries was identified from recent systematic reviews and meta-analyses. The relations were weakest for total sugars and most consistent for dietary sources corresponding to free sugars (including sugars added to and in fruit juices). Consideration of these health outcomes suggests that the emphasis for intake monitoring, public health guidance, and consumer communication should be on free sugars. However, at present, the adoption of free sugars for these purposes would also carry challenges related to implementation, including consumer understanding, consensus on specifications, and current (labeling) regulations.

## Introduction

There is consistent guidance to the public to reduce or limit intakes of sugars, which has been accompanied by public health policies and commercial action (e.g., product reformulations) intended to help achieve this goal. In 2015, for example, major evidence-based risk assessments with quantitative recommendations for sugars intakes were published by 3 major independent authorities: the WHO (1), the Scientific Advisory Committee on Nutrition (SACN) (2) in the United Kingdom, and the Dietary Guidelines Advisory Committee (DGAC) (3) in the United States. The recommendations and their quantitative basis are briefly summarized in **Table 1**. These indicate a relatively good consensus where the same outcomes were assessed, although the quantitative recommendations have different derivations. Furthermore, although WHO and SACN make their recommendations on “free” sugars, the DGAC recommendation is for “added” sugars. This variation is also apparent in recommendations from other international authorities (4, 5). It is, however, notable that none of these organizations base their recommendations on “total” sugars, although globally that is most commonly used for labeling and informing consumers about the sugar contents of foods and beverages.

**TABLE 1:** Quantitative recommendations of the WHO, SACN, and DGAC and the evidence considered as the possible basis for these recommendations^1^

		Evidence basis considered				
Author group,	Quantitative	Energy	Weight		Dental	
year (reference)	recommendation	intake	gain	Diabetes	caries	Other
WHO, 2015 (1)	<10% of energy from free sugars (<5% of energy from free sugars as “conditional” recommendation)	Not assessed	✓	Not assessed	✓^2^	None
SACN, 2015 (2)	≤5% of energy from free sugars	✓^2^	✓ (only in children)	✓ (for SSBs)	✓	Many other outcomes considered; none contributed toward the quantitative recommendation
DGAC, 2015 (3)	≤10% of energy from added sugars	Not assessed	✓	✓ (for SSBs)	✓	Increased risks of stroke, hypertension, and coronary heart disease and adverse impact on diet quality^2^

^1^DGAC, Dietary Guidelines Advisory Committee; SACN, Scientific Advisory Committee on Nutrition; SSB, sugars-sweetened beverage; ✓, adverse association or effect identified.

^2^Outcome used as primary basis for deriving the specific quantitative recommendation.

## Total, Added, or Free: How Do They Differ?

Broadly accepted definitions of total, added, and free sugars are given in **Table 2** (5–8). The differentiation between added and free sugars is particularly relevant. All added sugars are also free sugars, and both exclude all of the naturally occurring sugars in dairy foods and in intact (fresh, cooked, or dried) fruit and vegetables. The key distinction between added and free sugars is that the latter includes all naturally occurring sugars in nonintact (i.e., juiced or pureed) fruit and vegetables. However, as noted by others (5, 7–9) and considered in further detail below, there still are some inconsistencies and gray areas to be resolved in defining added and free sugars for research, monitoring, and labeling, as well as for public health and policy uses.

**TABLE 2:** Generally accepted definitions of total, added, and free sugars^1^

	Definition
Total sugars	All mono- and disaccharides present in food, derived from any source. In practice, this primarily consists of sucrose (table sugar), fructose, glucose (dextrose), and lactose (milk sugar). “Sugar” usually refers specifically to sucrose (table sugar) but sometimes refers to all sugars.
Added sugars	Sugars added to foods during processing or preparation (e.g., brown sugar, corn sweetener, corn syrup, dextrose, fructose, glucose, sucrose, high-fructose corn syrup, honey, invert sugar, lactose, maltose, malt syrup, molasses, raw sugar, and naturally occurring sugars that are isolated from a whole food and concentrated so that sugar is the primary component, e.g., fruit juice concentrates). “Added sugars” excludes naturally occurring sugars present in intact fruit, vegetables, or dairy products or in juiced or pureed fruit and vegetables.
Free sugars	All mono- and disaccharides except those that are naturally occurring and present in whole (intact, cooked, or dried) fruit and vegetables or dairy products. “Free sugars” includes all sugars added by the manufacturer, cook, or the consumer as well as sugars that are naturally present in juiced or pureed fruit and vegetables.

^1^From references 5–8.

The existence of these different ways of classifying sugars in foods and beverages in authoritative dietary guidance and nutrition communication implies that the distinctions are deemed to be physiologically relevant. The WHO, SACN, and DGAC all reported adverse relations of free or added sugars with various specific health outcomes, whereas the DGAC additionally concluded that desired food and nutrient intake patterns would be adversely affected or difficult to achieve at higher intakes of added sugars (3). An adverse association of added but not total sugar intakes with diet quality was also confirmed in a recent systematic review (10). Overall, however, the justification for expert groups to base their analyses and recommendations on free compared with added sugars is not explained in any explicit detail in the respective reports.

Sugar molecules are, of course, chemically and biologically indistinguishable by source, and therefore any physiologic differentiation between these classes must arise mainly from effects of the matrix in which the sugars are found. For example, it has often been shown that the acute metabolic impact is lower and satiety effects greater for intact fruit than for the comparable fruit juices, the latter having effects more similar to other sugars-sweetened beverages (SSBs) (11–16). Thus, variation in the nature of the food (or beverage) matrix clearly can result in differences in the postingestive effects of sugars consumed from these sources.

Given the known effects of the matrix in which sugars are found, it is reasonable to consider how total, added, and free sugars might differ in their relations with health outcomes. By definition, “total sugars” does not differentiate among sugars derived from different sources and implicitly presumes that sugars from all sources would have similar physiologic effects. Focusing on added sugars may partly resolve this, although the term describes only how sugars got into the foodstuff, not the nature of the matrix. Because the differences between total, added, and free sugars mainly pertain to the inclusion or exclusion of sugars naturally occurring in dairy products and fruit (intact or juiced), evidence relating to sugars from these sources is critical to determine whether and how the different classifications of sugars might be relevant for public health.

## Relations of Different Sugar Sources with Health Outcomes: Energy Balance, Diabetes, and Dental Caries

The research evidence base for sugars often does not explicitly define or differentiate exposures sufficiently to allow for direct analysis of total compared with added or free sugars. These limitations arise from the way that dietary data are collected and coded, the lack of standardization in the categorization of sugars in foods or beverages (especially fruit-based products), and differences between nutrient databases (5). The literature on sugar-health relations is dominated by research on total sugars (as a nutrient) and on commercially manufactured soft drinks, intakes of which are relatively easily defined and measured and that make a substantial single-category contribution to total, added, and free sugar intakes in many regions.

It is, however, also possible to look at health relations with the main food and beverage components that largely account for distinctions among total compared with added or free sugars, particularly intact fruit compared with fruit juice, and dairy products. By focusing specifically on these and drawing primarily on evidence from recent systematic reviews and meta-analyses, it is possible to evaluate whether the distinction between total, added, and free sugars is likely to be relevant, and which term most consistently aligns with relations with health outcomes.

None of the recent evidence-based expert assessments provide recommendations in terms of total sugars (1–3), although this is the component most widely communicated to consumers via nutrition labeling. For risk of weight gain and diabetes, a large body of systematic reviews and meta-analyses consistently report neutral or beneficial relations of dairy products and intact fruit and vegetables with risks of weight gain, obesity, or diabetes (10, 17–32). Systematic reviews and meta-analyses evaluated by SACN (2) and DGAC (3) as well as the German Nutrition Society (33) also led these expert panels to conclude that there was no significant association of diabetes risk with total sugars intakes. On the basis of these analyses, it is apparent that neither total sugars nor the sugar sources (dairy, intact fruit) that differentiate total sugars from added or free sugars are found to be significantly related to these health outcomes.

It is therefore relevant to focus attention more specifically on the evidence related to free compared with added sugars, which are primarily differentiated by the inclusion or exclusion, respectively, of naturally occurring sugars in fruit juices and purees. For outcomes related to energy balance, prospective cohort data fairly consistently indicate that fruit juices carry a risk of greater energy intake and body weight as do other predominantly sugars-based beverages (27, 34–37), although a recent meta-analysis reported that the association effect size was small and significant only in younger age groups (38). These results for fruit juices contrast with the consistent absence of adverse relations in analyses of intact fruit and vegetables, as noted above. The meta-analyses by Crowe-White et al. (34) of 100% fruit juice in children are enlightening here, because they show that the adverse association of fruit juices with anthropometric outcomes is only apparent from studies that did not adjust for energy intake. This would be consistent with the view that sugars-based beverages (including fruit juices) increase the risk of weight gain via effects on energy intake (2), although Auerbach et al. (38) found that effect sizes were little influenced by energy adjustment. These data overall, however, support the differentiation of sugars in intact fruit compared with fruit juices and thus the health relevance of free (compared with added) sugars.

For risk of diabetes, evidence supporting a differentiation between added and free sugars is limited and mixed. On the basis of analyses of data from 3 large cohorts (∼3.5 million person-years), Muraki et al. (39) reported an adverse association of fruit juice (but not whole fruit) with incident diabetes. Imamura et al. (40) reported that a significant association of diabetes risk for fruit juice was present but was weaker and “unstable” relative to other categories of SSBs, whereas Xi et al. (41) reported a significant association of diabetes with sweetened fruit juice but not “whole” fruit juice. However, in their systematic review and meta-analysis of fruit juice intervention trials, Wang et al. (42) found no significant effects of fruit juice on fasting glucose or insulin, glycated hemoglobin, or insulin resistance. It is unclear whether other components of some whole fruit juices may, in part, mitigate the contribution of their free sugars content to diabetes risks, leaving mixed evidence for free relative to added sugars.

Last, it is not clear if distinctions between total, added, or free sugars are relevant for the risk of dental caries, because all sugars (and starches) are recognized as potentially cariogenic. On the basis of a narrative review of cohort studies, SACN (2) concluded that there was a significant adverse association between total sugars intakes and dental caries but did not draw conclusions on any specific dietary sources other than the general category of SSBs. The systematic review and meta-analyses (43) underpinning the WHO (1) guidelines focused on free sugars, and therefore only included fruit in the context of juice, analyzed together with other free sugars sources. On the basis of the available reviews and reports, there does not seem to be a clear basis to focus on added rather than on free (or total) sugars with regard to dental caries risk. Accordingly, there is generally little differentiation made between fruit juices and other sugars-based beverages in guidance for preventing tooth decay (44–46). Evidence from a recent systematic review and meta-analysis indicates that (acidic) fruit juices have an adverse association with tooth erosion, which is significant although less robust than for soft drinks, whereas a beneficial association was found for milk and yogurt (47). Although a role of pH cannot be excluded, these results overall would tend to align with a free sugars basis for recommendations.

## Total, Added, or Free: Does It Matter?

Taken together, the evidence summarized here underscores the point that, although the molecules are the same, there is an important differentiation in the relations of health risks to sugars delivered by different dietary sources, which supports distinctions between total, added, and free sugars. For energy intake and weight gain, it seems reasonable to conclude that concern should be directed at free (compared with total or just added) sugars. For diabetes, it is clear that total sugars fails to distinguish the exposures associated with adverse risks, although the evidence is mixed as to whether the greater diabetes risk reported for manufactured SSBs (reflecting added sugars) is also shared by pure fruit juices (reflecting free sugars). For dental caries, there is also some evidence favoring an emphasis on free sugars, and no clear justification for limiting this only to added sugars. The evidence does not implicate the naturally occurring sugar (lactose) in dairy products in any of these outcomes, although assignment of any relations to the lactose component specifically would in any case be difficult.

Overall, considering the nature of sugar sources and relevant mechanisms, the totality of this evidence on health risks favors free sugars as the preferred focus of attention for public health intervention. Although whole fruit juices comprising the totality of the edible fruit portions can be a meaningful source of beneficial nutrients (48), they are also a source of free sugars. Further consideration may be given to how the total composition of some juiced or pureed fruit and vegetable products might influence the health impact and communication of their sugar contents. At present, however, advice to consider sugars in fruit juices as part of addressing free sugars intakes is also consistent with other general dietary guidance, especially for pediatric populations (46, 49–52).

## Challenges for Free Sugars

Solely on the basis of relations with health outcomes, free sugars may be seen as the most relevant basis for sugars-related public health action. However, there are important challenges to be addressed in implementing free (compared with total or added) sugars in regulation and public health guidance. These mainly fall under 3 headings, as outlined in the following sections.

### Definition and measurement

Globally accepted definitions and methods of assessment of sugars are needed for consistency in intake assessments, monitoring, labeling, enforcement, and consumer communication.

 The definition and chemical analysis of total sugars are straightforward and unambiguous. In contrast, the various definitions of added sugars begin to highlight the inconsistencies in the classification of sugars from certain ingredient sources for assessing compliance with recommendations and labeling requirements (5, 8). This is further complicated by a lack of accepted analytical methods to quantify added sugars and to discriminate them from total sugars in a product. Added sugars labeling places the onus on manufacturers to ensure that they know and document the added sugars content of all the ingredients that they use in their products, so the total value for added sugars can be calculated (8). Calculating the value to declare for products in which added sugar is lost during manufacturing (e.g., through fermentation or caramelization) would be even more of a challenge. Enforcement bodies will have to rely on manufacturers’ records for compliance checks, because it is not possible to distinguish between added and total sugars by analytical techniques. The inability to distinguish between added and total sugars analytically also makes adulteration of products with added sugars more difficult to detect (e.g., if a fruit juice is claimed to be 100% juice but has added sugar to improve the flavor or to reduce the cost of production).

For free sugars, there are further challenges in the development of a completely consistent and scientifically justified definition. In addition to the measurement issues for added sugars highlighted above, issues arise with processed forms of fruit and vegetables. The sugars from these clearly are not added, but after processes such as chopping, cooking, sieving, etc., these sugars will be present within a heterogeneous mix of liquids and particles, and thus along a continuum between clearly intact and clearly free sugars sources, with variable amounts of other plant components. Within this, it is uncertain where or how the boundary between “intact” and free should be set. The sugars naturally present in vegetables in soups or sauces are an example of this. Some of these issues have been considered by SACN for the United Kingdom (6), but a final view has not been published as of this writing, and we are not aware of similar efforts in any other regions. Given the evidence for free sugars as the most relevant component for health outcomes, there is a clear need for a globally accepted standard for definition. This, in turn, will benefit from research that more finely resolves structure-function relations for sugars from different sources and states of processing as a basis for more evidence-based criteria.

### Labeling regulations

At present, almost all mandatory and voluntary labeling of sugars in foods is based on total sugars only. Many countries follow the current Codex Alimentarius Guidelines on Nutrition Labeling (53), which require the labeling of total but not added (or free) sugars. When a revision of those guidelines was undertaken in 2010, the Codex Committee on Food Labeling supported the labeling only of total rather than added sugars (54) on the basis of the following: *1*) their view that the body cannot differentiate between added and total sugars, *2*) the absence of analytical differentiation needed for enforcement, and *3*) the suggested importance of total sugars for certain populations, including people with diabetes. In many regions with mandatory nutrition labeling of total sugars, voluntary labeling of added or free sugars currently would not be permitted.

In May 2016, the United States became the first country with regulations for supplemental sugars labeling that mandate added sugars labeling as a subgrouping of total sugars on the Nutrition Facts panel on foods and beverages. The US FDA justified this mandatory declaration on the basis of the need to provide consumers with the information needed to construct a healthy dietary pattern that is low in added sugars, rather than an independent relation between added sugars and the risk of chronic disease. The final rule also includes the requirement for a “percent daily value” to help consumers to determine if a product is high or low in added sugars and the relative contribution that a serving of that product will provide toward their diet (8, 55).

Health Canada recently published a consultation document on front-of-package nutrition labeling, which proposes the wording “high in …” for foods high in sodium, sugars, and saturated fats. For sugars, they propose that the label would apply to foods containing free sugars including fruit juice and that unsweetened fruit, vegetables, and dairy products would not be required to carry a front-of-package sugars label (56). This is the first regulatory authority to propose nutrition labeling on the basis of free sugars.

### Consumer understanding

A key issue for achieving public health goals is whether consumers would understand the relevance of different sources of sugars in the diet and be able to use this information to choose food products with lower amounts of free sugars. Consumers should be familiar with the current labeling of total sugars because this has been in place in many countries for many years, and evidence indicates that consumers can use this in comparing products and making choices more in line with dietary guidance (57, 58). The question, then, is whether they can use and benefit from the addition of new information on added or free sugars.

Kyle and Thomas (59) reported that consumers believe labeling of added sugars will be more helpful than confusing. However, the limited research carried out so far on consumer understanding of the labeling of added sugars has generated conflicting results (5). Research by the US FDA (60) showed that added sugars declarations increased the ability of some participants to identify products with less added sugars and to determine the quantity of added sugar in a food. Nevertheless, both that research and that of Laquatra et al. (61) also found that the declaration decreased the ability of some participants to correctly identify the quantity of total sugars in a food. A common error was for participants to overestimate the quantity of total sugars in the product by summing the product's total and added sugars. In contrast, research in Canada by Vanderlee et al. (62) indicated that the disclosure of added sugars and the inclusion of a percentage of Daily Value would have positive effects on consumer awareness and understanding of added sugars in packaged food products. Added sugars labeling led to more consumers identifying products that contain added sugars and the percentage of DV helped consumers to identify products that contain high amounts of added sugars.

There is little published research related to consumer understanding of free sugars specifically. Tierney et al. (9) recently reported that consumers in Northern Ireland had a very low awareness of the WHO guidelines (1), and a classification task suggested confusion in recognizing dietary sources of “added/free” sugars, results that reiterate the need to educate and inform consumers. Added sugars may be more intuitive and more familiar for consumers in some countries, yet if guidance is to be based on relations with health risks, it is arguably free sugars that should be the consistent focus of communication. In practice, the main difference between these for public health is the recognition and use of the term “free sugars” itself and greater attention being given to the intake of sugars from fruit juices and purees. This would require efforts to help consumers understand the concept and sources of free sugars, their relevance for a healthy diet, and how to find and use this information to make informed choices. However, unless regulations also change to allow the labeling of free sugars, it will be a challenge for consumers to know the free sugars content of any food or beverage product from the nutrition information given on the package.

International policy recommendations from the WHO (1) and the Pan-American Health Organization (63) are based on free sugars. Other national and regional authorities assess and communicate on total, added, or free sugars (4). Much of the scientific and regulatory debate focuses on added sugars or fails to make any clear distinction at all. To facilitate consumer education, it would be helpful for the international expert community to take care to use the specific, intended terminology (total, added, or free, where appropriate) in a consistent way. Although the evidence on health effects suggests that expert attention should focus on free sugars consumption, the additional considerations noted above may influence decisions on how this concept is applied in consumer communication.

## Conclusions

Relative to total and added sugars, the term “free sugars” best conveys the nature and sources of dietary sugars that are most consistently related to risks of positive energy balance, and that are also associated with diabetes and dental caries. Free sugars are also the basis for recent international policy recommendations on sugars (1). However, successful implementation of free sugars as a basis for intake assessment, public health guidance, and consumer communication would benefit from a consistent and globally harmonized specification, wider recognition and use of the term in the expert and regulatory communities, and assurance that the concept is understood by consumers.

## Notes

Perspectives articles allow authors to take a position on a topic of current major importance or controversy in the field of nutrition. As such, these articles could include statements based on author opinions or points of view. Opinions expressed in Perspectives articles are those of the author and are not attributable to the funder(s) or the sponsor(s) or the publisher, Editor, or Editorial Board of *Advances in Nutrition*. Individuals with different positions on the topic of a Perspective are invited to submit their comments in the form of a Perspectives article or in a Letter to the Editor.

Support for the preparation and publication of this review came from Unilever.

Author disclosures: DJM and EMW are employees of Unilever, a manufacturing company with a portfolio that includes sugar-containing foods and beverages. DJM is a member of the UK Scientific Advisory Committee on Nutrition (SACN) and was a member of the SACN Carbohydrates Working Group that produced the SACN carbohydrate recommendations cited in the text. The views expressed here are those of the authors and not necessarily shared by these other organizations.

**Abbreviations**
DGAC: Dietary Guidelines Advisory Committee
SACN: Scientific Advisory Committee on Nutrition
SSB: sugars-sweetened beverage
